# Improved BioGents® Sentinel trap with heat (BGSH) for outdoor collections of* Anopheline* species in Burkina Faso and Mali, West Africa

**DOI:** 10.1186/s13071-020-04527-y

**Published:** 2021-01-28

**Authors:** Amadou Guindo, Patric Stephane Epopa, Sidy Doumbia, Abdoul-Azize Millogo, Brehima Diallo, Franck Adama Yao, Bilkissou Yagoure, Frederic Tripet, Abdoulaye Diabate, Mamadou B. Coulibaly

**Affiliations:** 1 Malaria Research and Training Centre, University of Bamako, Bamako, Mali; 2 Institut de Recherche en Sciences de la Santé, Bobo-Dioulasso, Burkina Faso; 3 Institut des Sciences des Sociétés, Ouagadougou, Burkina Faso; 4 Centre for Applied Entomology and Parasitology, School of Life Sciences, Keele University, Staffordshire, UK

**Keywords:** *Anopheles gambiae* (*s.l.*), Malaria, Outdoor biting, Exophagy, Odour-baited traps, Counter-flow traps, Host-finding, Heat attraction, Monitoring, Surveillance

## Abstract

**Background:**

Since the late 1990s, malaria control programmes have relied extensively on mass bednet distribution and indoor residual spraying. Both interventions use pesticides and target mosquitoes coming indoors either to feed or to rest. Unfortunately, these intensified vector control campaigns have resulted in mosquito populations with high levels of resistance to most of the chemical compounds used against them and which are increasingly exophagic and exophillic, hence difficult to monitor indoors. Consequently, there is an urgent need for novel tools to sample outdoor anopheline populations for monitoring interventions and disease surveillance programmes.

**Methodologies:**

In this study, we tested several modifications and configurations of the BioGents® Sentinel (BGS) trap, designed with the aim to increase its efficacy for sampling malaria vector species. Traps were used with chemical attractants and CO_2_, and the impacts of trap position, trap colour contrast combination and the addition of a heat source were tested in two studies conducted in the Sudano-Sahelian region of Burkina Faso and Mali.

**Results:**

The results show that of all the configurations tested, the addition of a heat source to the BGS trap with the original colour combination and an upward positioning resulted in a 1.8- and 5.9-fold increase in host-seeking *Anopheles gambiae* (*s.l.*) females in the experiments performed in Burkina Faso and Mali, respectively. BGS with heat traps, referred to as BGSH traps, captured *An. gambiae* (*s.l.*), *An. pharoensis*, *An. coustani*, *Culex* and *Mansonia* spp. Importantly, the results suggest that their efficacy does not depend on the close proximity of nearby hosts in houses.

**Conclusions:**

The results suggest that BGSH traps can be an effective scalable tool for sampling outdoor anopheline vector populations. Further developments enabling CO_2_ and heat generation for longer periods of time would further improve the trap’s versatility for large-scale surveillance programmes. 
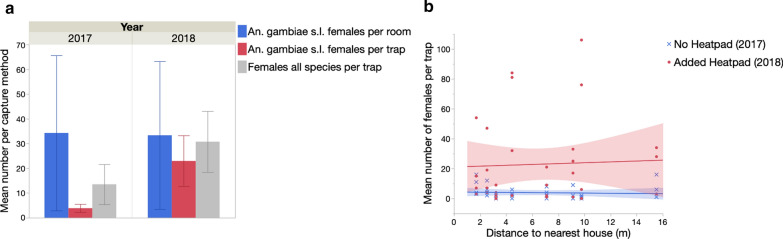

## Background

Since the late nineties, the death toll attributed to malaria in sub-Saharan Africa has decreased from over 2 million to less than half a million per year [1]. Much of this progress is attributed to the control of anopheline vector populations through a policy of mass distribution of long-lasting impregnated bednets (LLINs) treated with pyrethroids [2]. In some settings, the mass distribution of LLINs is combined with campaigns of indoor residual spraying (IRS). However, faced with increasing pesticide exposure indoors, populations of the *Anopheles gambiae* and *An. funestus* complexes, the main malaria vectors in Africa, have undergone many genetic, physiological and behavioural changes that have enabled them to remain a threat.

Behavioural changes, whether due to the evolution of novel behaviour or to existing behavioural plasticity and resilience, have been reported in many settings. The most commonly observed behavioural change is a shift from biting and resting indoors to increasingly feeding and resting outside homes [3–5]. In some locations, vectors bite more frequently earlier in the evening or at dawn when humans are not protected by their bednets [6–8]. The result of such behavioural changes, in combination with widespread genetic resistance to pesticides, is that in many settings malaria transmission cannot be stopped with currently available scalable vector control tools [9]. Another consequence of these changes is that mosquito surveillance and monitoring tools that are mostly geared to monitoring indoor biting vectors are often ineffective or not scalable for outdoor use [9].

The effective sampling of female mosquitoes that are seeking to feed on human hosts is a crucial tool for medical entomologists as it provides vital information about the vector species present, their relative abundance and their rate of infection with *Plasmodium*, the malaria parasite [10, 11]. Traditionally, this information was collected via human landing catches (HLC); however, this strategy raises ethical concerns as it potentially exposes human collectors to malaria and increasingly to emerging and resurging arboviral infections [12, 14]. Spray catches, indoor aspiration and resting traps are other methods that work well indoors, but these are less rewarding and difficult to standardize outdoors [9–11, 13]. There are a variety of light traps or odour-baited traps that work well inside or in close proximity to houses as they take advantage of the attraction of mosquitoes to humans. However, when placed further away from habitations, their efficacy drops dramatically [13, 15, 16]. There is therefore a strong need for novel scalable sampling tools to enable the monitoring of outdoor biting anopheline vector communities.

The BioGents® Sentinel (BGS) trap (BioGents AG, Regensburg, Germany) has proven to be a very important tool for surveillance programmes focusing on Aedine mosquitoes responsible for the transmission of emerging and re-emerging viral infections, such as dengue, chikungunya and Zika. These traps use a counter-flow geometry design, combined with colour contrast and chemical attractants with great efficacy. To attract anophelines, a source of carbon dioxide (CO_2_) is recommended, but despite that addition to the BGS trap, results from different settings have been often poor. In Brazil, BSG traps proved effective for sampling *An. darlingi*, but they required modifications in colour contrasts and performed better when facing downwards in an inverted position [17]. In Africa, the traps were shown to be more effective than U.S. Centers for Disease Control and Prevention (CDC) light traps outdoors overall but captured low numbers of the malaria mosquito *An. gambiae* (*s.l.*) [18]. In Tanzania, semi-field studies and field studies showed that positioning traps downwards improved catch rates compared to the other configurations [19, 20].

In this study conducted in the Sudano-Sahelian zone of West Africa, we tested the effects of trap position, different colour contrasts and the addition of a heat source on the efficacy of BGS traps for capturing females of *An. gambiae* (*s.l.*), the main vector of malaria and also a vector of lymphatic filariasis [21–25]. The performance of the BGS traps with heat (BGSH) is discussed in the broader context of research and development for scalable standardized sampling methods for monitoring exophagic and exophillic malaria vector populations and measuring the impact of vector control programmes aiming to curb malaria transmission.

## Methods

This study was carried out during the period of 2016–2018 in Burkina Faso and Mali, two countries in the West African region. A first study was carried out in Burkina Faso in September 2016 to assess the benefit of adding a heat source to BGS traps to catch anopheline mosquitoes in a Sahelian context. A second study was conducted in Mali in 2017 and 2018 to further assess the impact of the heat source in combination with several colour combinations for field monitoring of *Anopheles gambiae* (*s.l.*) and potentially other anopheline species.

### Experiment in Burkina Faso

#### Study site

**Fig. 1:**
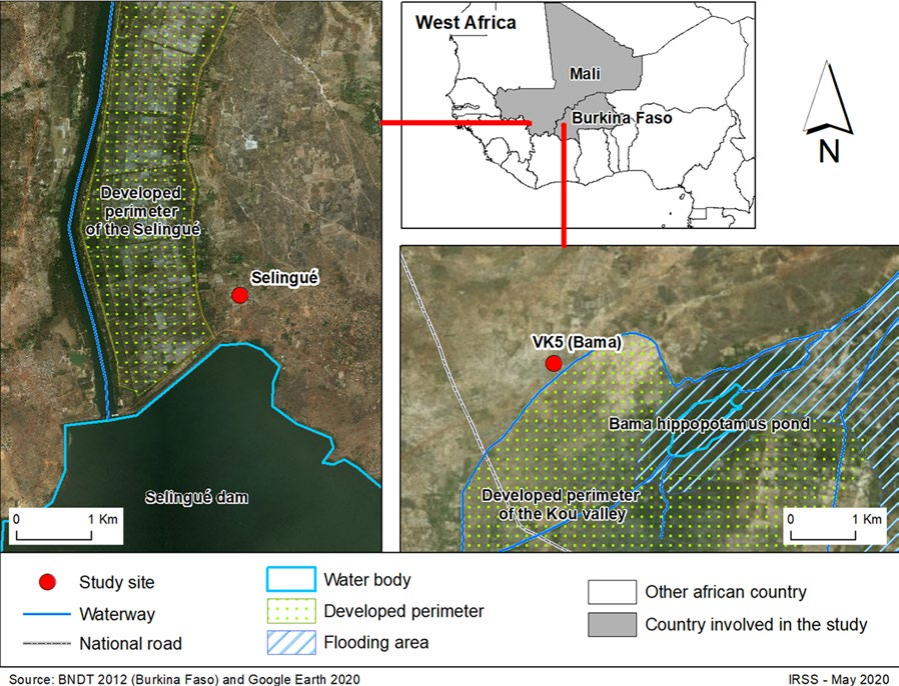
Location of the study sites for the Burkina Faso 2016 and Mali 2017–2018 experiments

In Burkina Faso, the survey was carried out during September 2016 (rainy season) at one of the IRSS (Institut de Recherche en Sciences de la Santé) research facilities located at village 7 in the Kou valley (VK7), in western Burkina Faso. The traps were placed outdoors around the IRSS mosquito ecology research facility (MERF; a mosquito semi-field containment facility) at a distance of about 200 m from the nearest inhabited house of the village. A group of seven villages are found in the Kou valley (Fig. 1a), located about 30 km north of Bobo-Dioulasso (11°23′14″N; 4°24′42″W). This rice-growing area of about 12 km^2^ and 4470 inhabitants is characterized by a mean annual rainfall of about 1200 mm [26]. In this region, the rainy season spans June to October and the dry season extends from November to May. The anopheline mosquito abundance is generally considered to be high in this region throughout the year, with a mean indoor biting rate of about 200 bites per person per night [27]. *Anopheles gambiae* (*s.l.*) mosquitoes, predominantly *An. coluzzii* followed by *An. gambiae*, are the major malaria vectors in the area, with the next most abundant vector being *An. funestus* (*s.l.*) [28].

#### Trap configurations

**Fig. 2:**
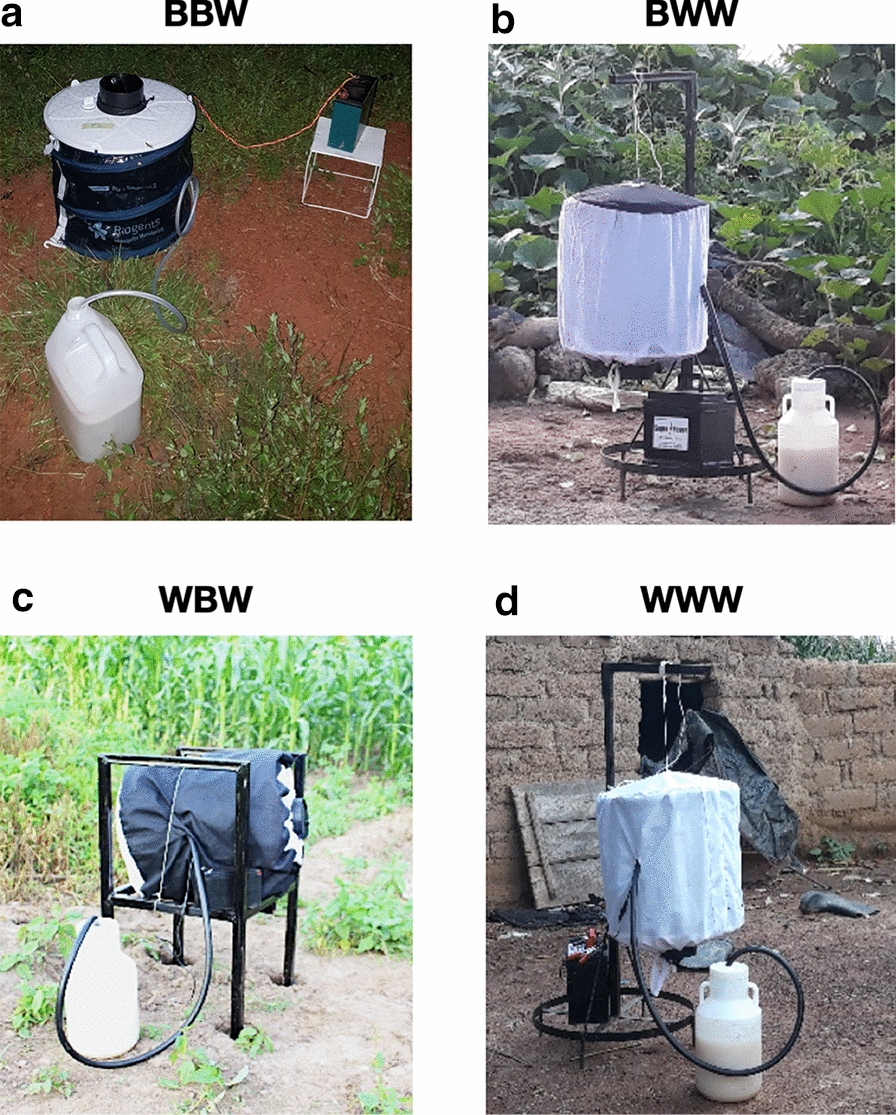
Examples of BioGents® Sentinel (BGS) trap (BioGents AG, Regensburg, Germany) configurations, with varying colour combinations and position. **a–d** All traps were used with a CO_2_ generator and, depending on the study, were positioned upwards, sideways or downwards.* BBW* Standard colour combination of very dark blue on the bottom and sides and white on the top, as shown in** a**. The Mali study also investigated three alternative contrast colour combinations: BWW (**b**), WBW (**c**), WWW (**d**)

A standard BGS trap consists of a cylindrical flexible bag (main body) with a diameter of 35 cm and height of 40 cm [29]. A collecting tube (diameter 14 cm, length 15 cm) located in the middle of the upper part of the cylinder is connected to an electric fan powered by a 12 V battery (7 Amp) producing inward aspiration, which draws approaching insects into the collecting tube and a netted catch bag. The standard colours of the BGS trap are a very dark blue on the bottom and sides and white on the top; this colour combination is subsequently referred to as ‘normal’ colours (BBW) throughout this article. A BioGents® Sentinel chemical lure (BioGents AG) that produces an attractive plume of odour was inserted into the lure compartment of the trap. A CO_2_ generator consisting of a 5-l jerrycan filled with 3 l of a water solution mixed with 300 g of sugar and inseminated with 20 g of yeast was also connected to the trap. CO_2_ generators were set up about 1 h before the start of each collection and produced CO_2_ for a period of about 16–18 h. A plastic connecting tube allowed the CO_2_ to flow from the jerrycan to the inside of the trap through a dedicated opening (Fig. 2).

##### Trap positions

The effect of the BGS trap positions (downwards or upwards) was assessed. Metal stands were used when needed to fix traps in the required position. All traps were positioned so that the airflow intake height was approximately 40 cm from the ground (Fig. 2).

##### Heat stimulus

To simulate human body heat, a Cura-Heat® heat pad (Kobayashi Healthcare Europe, Chiswick, London, UK) was added to the traps. The heat pads were placed inside the BGS trap and stuck onto the interior wall of the trap. New heat pads produce heat at approximately 36 °C for approximately 12 h; hence new pads were used for each collection night.

#### Experimental procedure

A completely balanced experimental design was conducted to explore the impact of trap position (downwards vs upwards) and of a heat source (heat pad added or not) on the efficiency of the BGS traps. Four traps, each with one of the four (2 × 2) combinations of position and presence/absence of heat source, were placed at four specific locations around the IRSS facility at the Kou Valley, at least 200 m from the nearest inhabited house and at least 500 m from the closest rice field. The trap position and heat combination were rotated among the four locations during six consecutive nights. BGS trap collections took place from 7 pm to 7 am the following morning.

After each collection, the BGS traps were moved to the field laboratory. The netted catch bags were collected and mosquitoes were transferred into a labelled petri dish for identification and recording. Collected mosquitoes from each BGS trap were sexed and identified morphologically in the field using a field stereomicroscope (Perfex Sciences® Zoom Pro., Perfex Sciences, Toulouse, France), counted and preserved in 80% ethanol. The total number of mosquitoes from *An. gambiae* (*s.l.*) and other anopheline and non-anopheline mosquitoes collected each day was used to estimate the trap configuration catch rate as a proxy measure of the trapping performance.

### Experiments in Mali

#### Study site

Two experiments were conducted during 2017 and 2018 in a hamlet (Village 20) located in the rice-growing area of Selingue Rural Development Office (ODRS), located 140 km south-east of Bamako, the capital of Mali (Fig. 1). The area is located in the south Sudanian savannah and has an annual rainfall of 700–1300 mm (Cinquième Enquête Démographique et de Santé du Mali records, 2012–2013). It is part of the irrigated zone of a hydroelectric dam built nearby. The rice paddies are located less than 500 m from the first houses of Village 20. This proximity has resulted in a very strong mosquito nuisance and particularly high anopheline densities [30]. Due to irrigated rice cultivation, the Selingue health zone is characterized by plurimodal transmission, with stable type malaria [31]. The main malaria vectors in this region are the members of the *An. gambiae* complex, *An. coluzzii*, *An. gambiae *(*s.s.*) and *An. arabiensis*, with a few *An. funestus* (*s.l.*).

#### Trap configurations

All BGS traps were used with a chemical lure and a CO_2_ generator (as described above). Different trap configurations were used to assess the effects of three trap colour contrast patterns that differed from the standard colour combination (BBW), three trap positions (upwards, downwards and sideways) and a heat source, on the number of Anopheline mosquitoes captured (Fig. 2).

##### Colour contrasts

Four colour combination patterns of the BGS trap were tested to identify the most effective contrast pattern for the capture of anopheles. Four different possible combinations of contrast between the bottom, main body and upper part of the trap were evaluated using black or white textile sleeves to cover different parts of the traps (Fig. 2). In addition to the standard trap colours (BBW), traps with a white bottom, black sides and white upper part (WBW), black bottom, white sides and white top (BWW) and white bottom, sides and top (WWW) were tested (Fig. 2a–d).

##### Trap position

The effect of the BGS trap position (backwards, upwards or sideways) was assessed. Metal stands were used when needed to fix traps in the required position. All traps were positioned in such a way that the airflow intake height was approximately 40 cm from the ground (Fig. 2).

##### Heat stimulus

To simulate human body heat stimulus, a Cura-Heat heat pad (Kobayashi Healthcare Europe) was added to the traps. In this experiment, the heat pads were stuck onto the exterior upper part outside of the trap. New heat pads were used on each collection day. The potential benefit of adding such a heat stimulus on trapping performance was assessed.

#### Experiment procedure

A before and after study approach was taken by performing two completely balanced randomized design experiments, one in 2017 and one in 2018 in August at the peak of the rainy season period.

In 2017, collections were made using standard BGS traps in four different colour combinations and two positions (downwards and sideways), but with no additional heat source (Table 1). Four collection rounds were made over four nights using eight traps each night. During each night, one trap with each of the eight BGS trap configurations described was installed in the surroundings of a house (approx.  2–16 m from the building). Trap collections took place from 06:00 pm to 06:00 am the next morning. The location of each trap configuration was alternated each day.

**Table 1:** BioGents® Sentinel trap configurations in the Mali study

Year	CO_2_	Lure	BGS colour combination^a^	BGS trap position	Heat
2017	Yes	Yes	BBW, BWW, WBW, WWW	Downwards, sideways	No
2018	Yes	Yes	BBW, BWW, WBW, WWW	Upwards, downwards	Yes

BGS, BioGents® Sentinel trap

^a^BBW, Standard colour combination of BioGents® Sentinel (BGS) trap of very dark blue on the bottom and sides and white on the top; WBW, BSG traps with a white bottom, black sides and white upper part; BWW, BSG traps with a black bottom, white sides and white top; WWW, BSG traps with white bottom, sides and top

In 2018, a similar experiment was carried out using the same methodology. This time, traps of the four different colour combinations and two positions (downwards and upwards) were used with an additional heat source (Table 1). This design allowed the assessment of the potential effect of heat source on the trapping performance of the BGS traps.

As a control for variation in mosquito abundance between years, indoor mosquito density in the village was measured in 2017 and 2018, using pesticide spray catches (PSC). One day after the four nights of BGS collections, ten houses were selected randomly within the study area and one room per house was selected for morning PSC collection (around 8 am).

After each BGS trap and PSC collection, the mosquitoes caught were transported to the field laboratory. All mosquitoes were sexed and identified morphologically to the genus level using a field stereomicroscope (Perfex Sciences® Zoom Pro.) and identification keys [32–35]. Anopheline samples were further keyed to the species level. The number of individuals of each category was recorded and samples were preserved in 80% ethanol.

### Statistical analysis

For both studies, the mean number of *An. gambiae* (*s.l.*) and other mosquitoes per trap per night (catch rate) was used for comparing BGS trapping performance and analysed using generalized linear models with Poisson distribution with over-dispersion. Post hoc pairwise comparisons of effects were performed using model contrasts. Proportions of females and males were analysed as binomial variable using logistic regression (Logit function), and subsequent pairwise comparisons of effects were tested using likelihood odds ratios. In all models, interactions between independent variables were tested but then removed using a step-wise approach to retain only those significant in the final models. All statistical analyses were performed using JMP 14 software (SAS Institute, Inc., Cary, NC, USA).

## Results

### Burkina Faso experiment

A total of 1316 *An. gambiae* (*s.l.*) females and 82 males were collected using the BGS traps, in addition to 686 *An. pharoensis*, 32 *An. coustani*, 31 *Culex* spp. and 1384 *Mansonia* spp. females (Table 2). All captured *An. gambiae* (*s.l.*) females were unfed except for one blood-fed and one gravid female.

**Table 2:** Total and mean numbers of female and male *Anopheles gambiae* (*s.l.*) and of females of other taxa in the Burkina Faso 2016 experiment

Heat pad	Trap position	N	*Anopheles gambiae* (*s.l.*) females		*Anopheles gambiae* (*s.l.*) males		*Anopheles pharoensis*		*Anopheles coustani*		*Cullex.* spp.		*Mansonia* spp.		All females	
			Sum	Mean (SD)	Sum	Mean (SD)	Sum	Mean (SD)	Sum	Mean (SD)	Sum	Mean (SD)	Sum	Mean (SD)	Sum	Mean (SD)
No	Downwards	6	196	32.7 (22.2)	25	4.2 (6.3)	57	9.5 (8.8)	7	1.2 (1.2)	13	2.2 (1.7)	281	46.8 (33.4)	554	92.3 (42.4)
No	Upwards	6	314	52.3 (20.3)	8	1.3 (1.5)	175	29.2 (15.8)	3	0.5 (0.5)	0	–	284	47.3 (29.3)	776	129.3 (54.9)
Yes	Downwards	6	234	39 (19.8)	26	4.3 (8.3)	52	8.7 (4.9)	14	2.3 (2.1)	15	2.5 (2.2)	283	47.2 (29.4)	598	99.7 (35.3)
Yes	Upwards	6	572	95.3 (34.2)	23	3.8 (4.4)	402	67 (48.3)	8	1.3 (1.2)	3	0.5 (1.2)	536	89.3 (40.4)	1521	253.5 (87.6)

*SD* Standard deviaiton, *N* total number of trap-nights

#### Effect of additional heat source and trap orientation

##### Mean catch rates of *An. gambiae* (*s.l.*) females

**Fig. 3:**
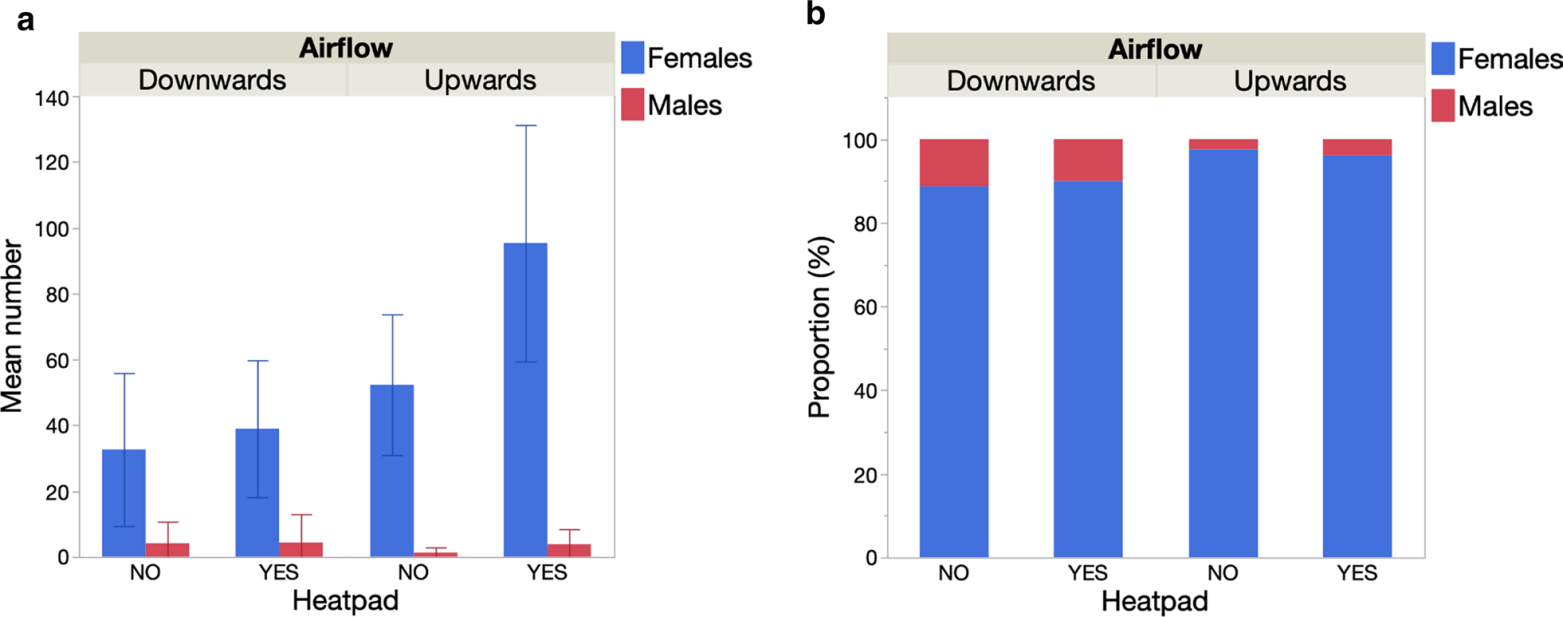
Effect of trap position and the addition of a heat stimulus on the mean number (**a**) and the proportion (**b**) of female and male *Anopheles gambiae* (*s.l.*) captured in the Burkina Faso study

Traps oriented upwards captured significantly more *An. gambiae* (*s.l.*) females than those positioned downwards (generalized linear model: likelihood ratio *χ*^2^ = 13.7, *P* = 0.001) (Fig. 3a). The addition of a heat source significantly improved the number of females captured (likelihood ratio *χ*^2^ = 5.7, *P* = 0.020) (Fig. 3a). The highest impact was measured in traps placed upwards, where catch rates increased by 1.8-fold.

##### Proportion of *An. gambiae* (*s.l.*) females and males

The addition of a heat source did not impact the proportion of males captured (logistic regression model: likelihood ratio *χ*^2^ = 0.01, *P* = 0.757). However, traps positioned downwards captured a significantly higher proportion of males than those placed upwards (likelihood ratio *χ*^2^ = 28.0, *P* < 0.001) (Fig. 3b).

**Fig. 4:**
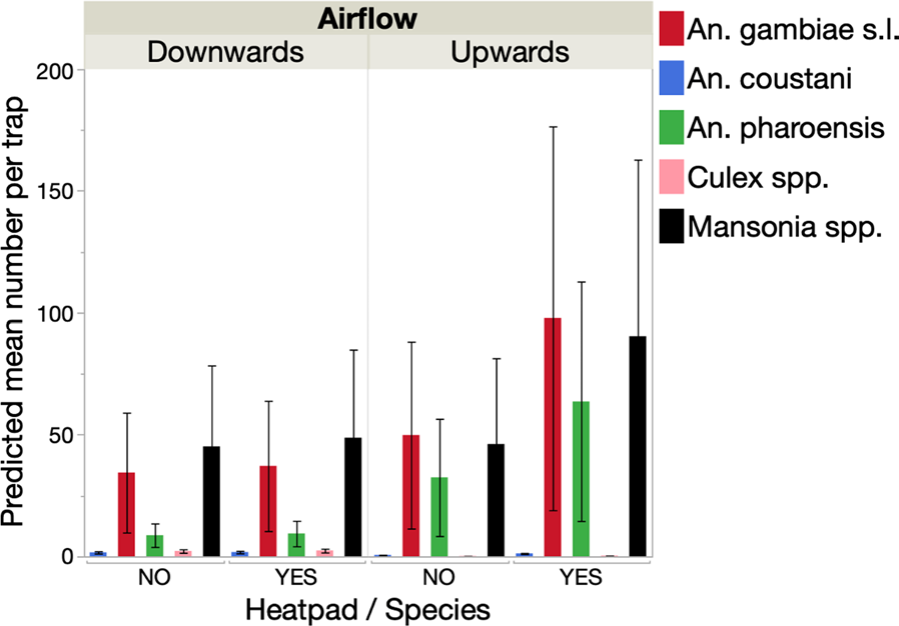
Effect of trap position and the addition of a heat stimulus on the number of females from different taxa captured in the Burkina Faso experiment

In the 2016 experiment, *An. gambiae* (*s.l.*) and* Mansonia* spp. females were equally abundant (model contrasts: *χ*^2^ = 0.64, *P* = 0.421), and captured in significantly higher numbers than *An. pharoensis* (model contrasts: *χ*^2^ = 25.06, *P* < 0.001), with the latter being significantly more abundant than *An. coustani* and *Culex* spp., both of which were both rare in this study (model contrasts: *χ*^2^ = 23.34, *P* < 0.001 in both cases) (Table 2). The addition of heat strongly impacted capture rates (Fig. 4; Table 3), with a particularly strong effect in the upwards trap orientation where it resulted in a twofold increase in capture rate(Fig. 4). There was no direct impact of trap position on catch rates, but trap position did interact significantly with the heat stimulus, as described above, as well as with species (Table 3).

**Table 3:** Generalized linear models of the effects of trap position, colour combination and species on the mean number of females from different species captured in Burkina Faso in 2016

Source	df	Likelihood ratio χ^2^	P value
Species	4	342.6	< 0.001
Heat	1	11.6	0.001
Trap position	1	0.04	0.854
Species × Trap position	4	22	< 0.001
Trap position × Heat	1	7.3	0.007

### Mali experiments

In 2017, a total of 124 female and 40 male *An. gambiae* (*s.l.*) individuals were collected, in addition to 308 females from other species (*An. pharoensis*, *An. coustani*,* Culex* or* Mansonia* spp.). In 2018, when a heat source was added to the BGS traps, a total of 735 female and 30 male *An. gambiae* (*s.l.*) individuals were collected, as well as 58 *An. pharoensis*, 147 *An. coustani*, 125 *Culex* spp. and 227 *Mansonia* spp. females (Table 4).

**Table 4:** Total and mean (SD) numbers of female and male *An. gambiae* s.l., and females of other taxa in the Mali 2017-2018 experiment

Year	Heat	Position	Colour	N	*An. ga* sl F^a^		*An. ga* sl M^b^		
					Sum	Mean(SD)	Sum	Mean(SD)	
2017	No	Downward	BWW	4	19	4.8 (4.3)	3	0.8 (1)	
2017	No	Downward	BBW	4	27	6.8 (2.2)	5	1.3 (2.5)	
2017	No	Downward	WBW	4	18	4.5 (7.7)	1	0.3 (0.5)	
2017	No	Downward	WWW	4	6	1.5 (1.7)	3	0.8 (1)	
2017	No	Sideways	BWW	4	17	4.3 (5.2)	9	2.3 (4.5)	
2017	No	Sideways	BBW	4	21	5.3 (7.4)	6	1.5 (3)	
2017	No	Sideways	WBW	4	8	2 (2.7)	10	2.5 (5)	
2017	No	Sideways	WWW	4	8	2 (2.8)	3	0.8 (1.5)	
2018	Yes	Downward	BWW	4	28	7 (7.1)	3	0.8 (0.5)	
2018	Yes	Downward	BBW	4	181	45.3 (28)	9	2.3 (1.7)	
2018	Yes	Downward	WBW	4	129	32.3 (36.9)	6	1.5 (1.7)	
2018	Yes	Downward	WWW	4	49	12.3 (14.7)	2	0.5 (0.6)	
2018	Yes	Upward	BWW	4	39	9.8 (12.5)	2	0.5 (1)	
2018	Yes	Upward	BBW	4	189	47.3 (43.5)	2	0.5 (1)	
2018	Yes	Upward	WBW	4	83	20.8 (36.9)	1	0.3 (0.5)	
2018	Yes	Upward	WWW	4	37	9.3 (6.9)	5	1.3 (1)	

*An. ph*^c^		*An. co*^d^		*Cu.* spp^e^		*Ma.* spp^f^		All Females	
Sum	Mean (SD)	Sum	Mean (SD)	Sum	Mean (SD)	Sum	Mean (SD)	Sum	Mean (SD)
0	0 (0)	0	0 (0)	0	0 (0)	7	1.8 (2.2)	26	6.5 (4.8)
0	0 (0)	18	4.5 (6.5)	9	4.5 (4.5)	6	1.5 (3)	60	15 (9.7)
0	0 (0)	2	0.5 (0.6)	1	0.5 (0.5)	3	0.8 (1)	24	6 (8.1)
0	0 (0)	0	0 (0)	2	0 (1)	1	0.3 (0.5)	9	2.3 (1.7)
0	0 (0)	50	12.5 (16.7)	7	12.5 (2.9)	35	8.8 (15.5)	109	27.3 (30.2)
0	0 (0)	24	6 (10.7)	76	6 (36)	5	1.3 (1.5)	126	31.5 (53.2)
0	0 (0)	3	0.8 (0.5)	4	0.8 (1.4)	18	4.5 (6.1)	33	8.3 (7.9)
0	0 (0)	9	2.3 (3.9)	6	2.3 (±3)	22	5.5 (6.8)	45	11.3 (13.7)
0	0 (0)	6	1.5 (1.7)	1	1.5 (0.5)	14	3.5 (2.6)	49	12.3 (7.3)
12	3 (4.8)	3	0.8 (1)	2	0.8 (0.6)	2	0.5 (0.6)	200	50 (26.9)
29	7.3 (8.8)	4	1 (±1.4)	1	1 (0.5)	10	2.5 (1.7)	173	43.3 (48.9)
8	2 (3.4)	3	0.8 (1)	1	0.8 (0.5)	15	3.8 (3.3)	76	19 (17.3)
1	0.3 (0.5)	1	0.3 (0.5)	2	0.3 (1)	1	0.3 (0.5)	44	11 (12.1)
5	1.3 (1.9)	12	3 (4.1)	5	3 (1.9)	57	14.3 (23.5)	268	67 (51.8)
0	0 (0)	7	1.8 (1.5)	7	1.8 (2.9)	17	4.3 (3.4)	114	28.5 (42.5)
3	0.8 (1.5)	5	1.8 (1.3)	1	1.8 (0.5)	14	3.5 (3.5)	60	15 (6.8)

^a^*Anopheles gambiae* s.l. female, ^b^*Anopheles gambiae* s.l. male, ^c^*Anopheles pharoensis,* ^d^*Anopheles coustani,* ^e^*Culex* species, ^f^*Mansonia* species

*N* total number of trap-nights

#### Effect of additional heat stimulus

**Fig. 5:**
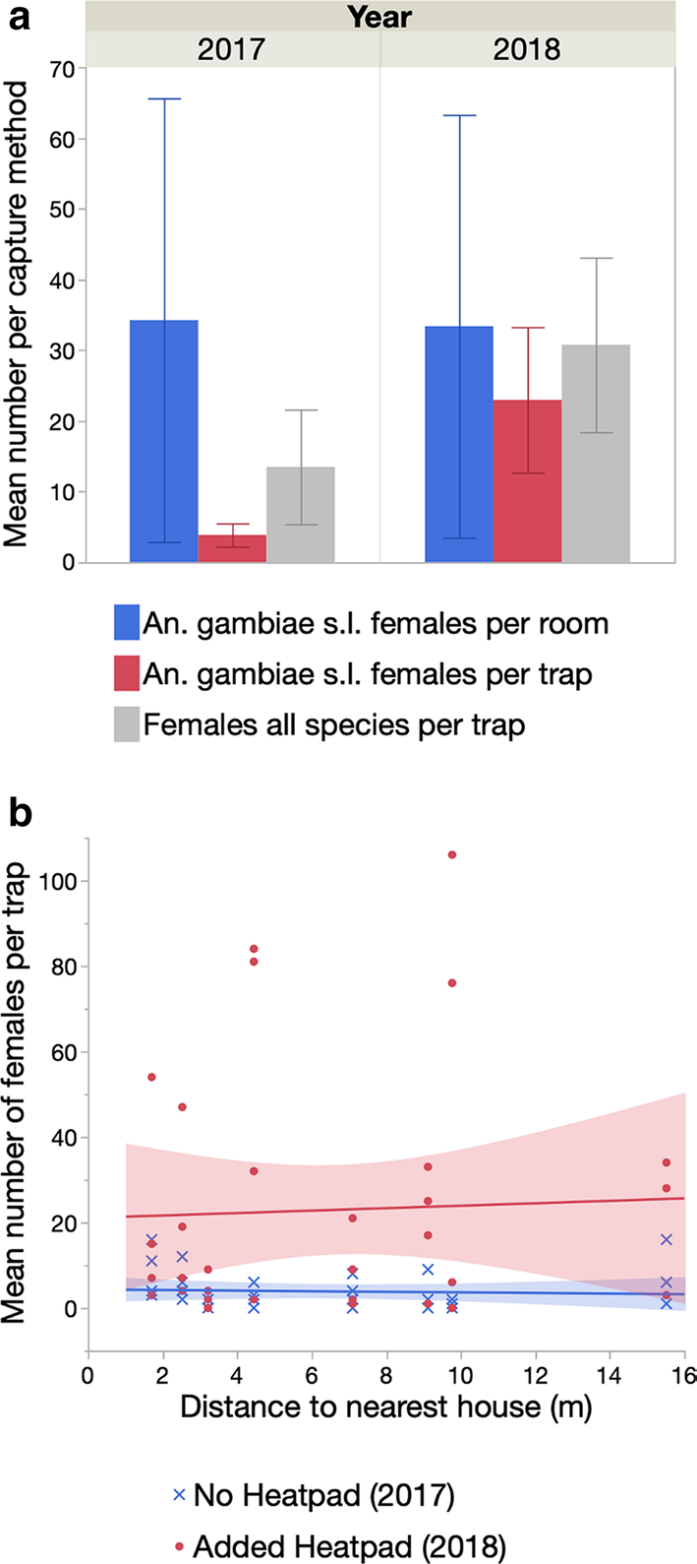
Mean number of *An. gambiae* (*s.l.*) females captured by indoor spray catches compared to the number of females captured with the BGS traps in the before-and-after experiment in Mali. **a** In 2017 BGS traps positioned outdoors with CO_2_ were used without heat stimulus while in 2018 a heatpad was added to improve catch rates. BGS traps captured host-seeking females of other mosquito species (see text for details). **b** The distance of the BGS trap from the nearest inhabited room had no effect on capture rates

Collections by PSC resulted in a mean catch of 34.3 (95% confidence interval [CI] 2.9–65.6) *An. gambiae* females per room in 2017 and 33.4 (95% CI 3.5–63.3) in 2018 (Fig. 5a). By comparison, BGS traps captured only 3.9 (95% CI 2.2–5.5) *An. gambiae* females per trap in 2017. However, in 2018, after adding a heat source to the trap, an average of 23.0 (95% CI 12.7–33.3) *An. gambiae* females were captured per trap, equivalent to a 5.9-fold increase (Fig. 5a). The number of females from other taxa captured also increased from 13.5 (95% CI 5.4–21.6) to 30.7 (95% CI 18.4–43.1) from 2017 to 2018 (Fig. 5a). A generalized linear model showed that *An. gambiae* female BGS catch numbers strongly and significantly increased with the addition of a heat source but were not affected by the distance of the trap to the nearest house and there was no interactive effect of heat source and distance to the nearest house (Table 5; Fig. 5b).

**Table 5:** Generalized linear model of the effect of an additional heat stimulus and distance to nearest house on the total number of female *An. gambiae* (*s.l.*) captured in the before-and-after study

Source	df	Likelihood ratio χ^2^	P value
Heat source	1	22.835	< 0.001
Distance BGS-room (m)	1	0.004	0.952
Distance BGS-room (m) × Heat source	1	0.089	0.765

#### *An. gambiae* (*s.l.*) female gonotrophic stages

**Fig. 6:**
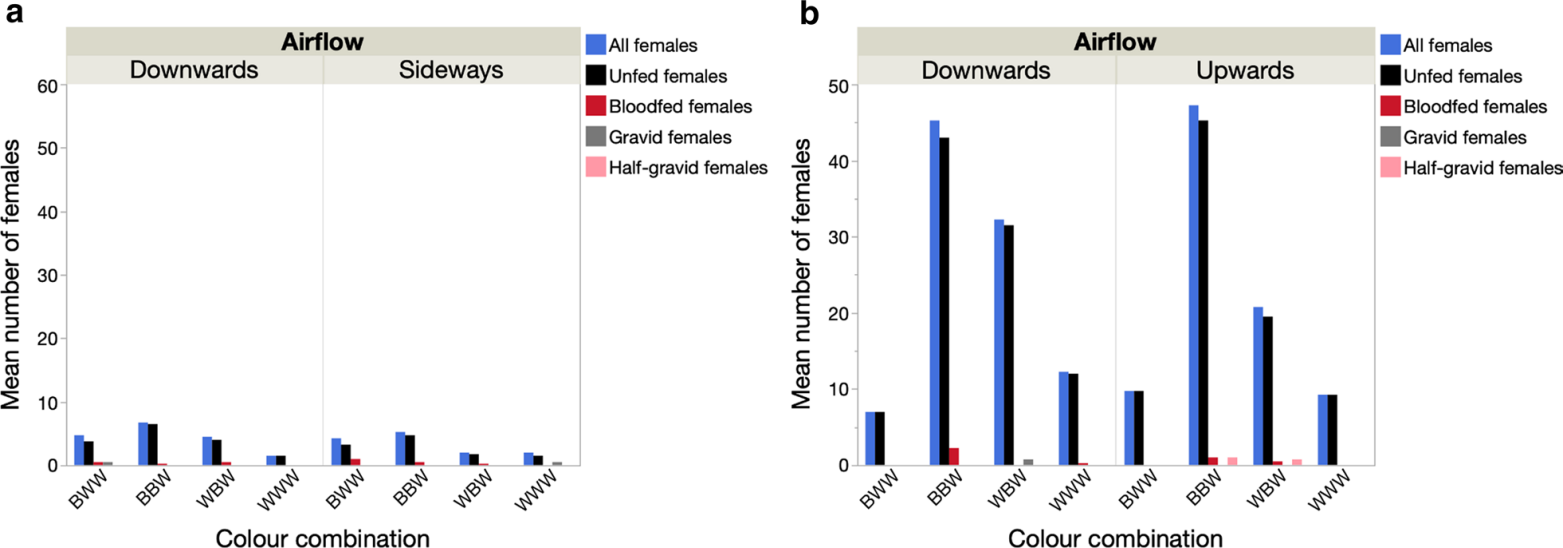
Effect of trap orientation and colour combination on the number of *An. gambiae* (*s.l.*) females at different gonotrophic stages in: **a** Mali 2017 BGS, **b** Mali 2018 BGS with heatpad

In 2017, unfed females were the most common category of females captured, representing 87.1% of samples (Wilcoxon signed-rank test: *n* = 32, *S* > − 209.5, *P* < 0.001 in all cases). Blood-fed females, while infrequent, were significantly more common than the very rare gravid and half-gravid females (*S  *= − 101.5*, P* = 0.006). In 2018, with the addition of a heat source, captures were further biased towards unfed *An. gambiae* (*s.l.*) females, which were the most common category captured, representing 96.5% of all females captured (Wilcoxon signed-rank test: *n* = 32, *S* > − 262.5, *P* < 0.001 in all cases) (Fig. 6).

#### Effects of trap orientation and colour combinations

##### Mean catch rates of * An. gambiae* (*s.l.*) females

**Fig. 7:**
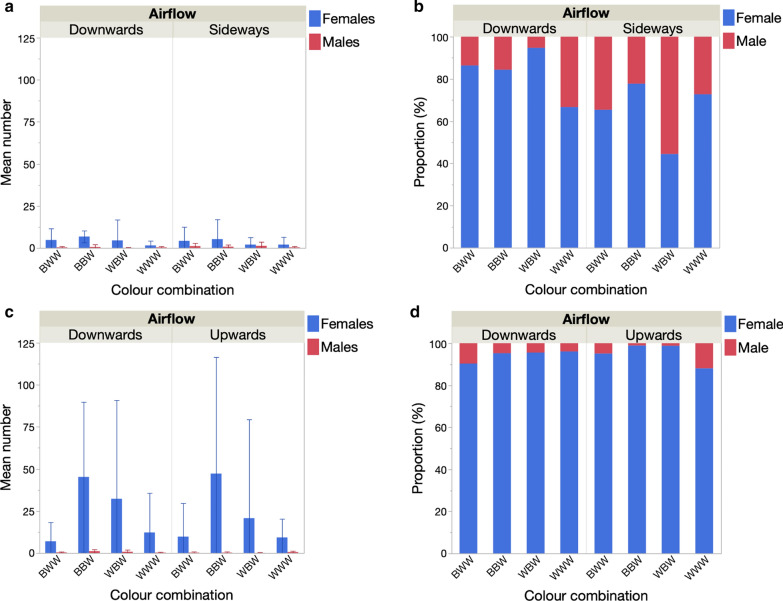
Effect of trap position and the addition of a heat stimulus on the mean number (**a**,** c**) and the proportion (**b**,** d**) of female and male *An. gambiae* (*s.l.*) captured in the Mali 2017 (top) and Mali 2018 experiments (bottom)

In either year, trap position (orientation downwards vs sideways and upwards vs downwards) had no significant effect on the mean number of *An. gambiae* (*s.l.*) females captured (Table 6, Fig. 7a, c). In 2017, there was no effect of trap colour combination on the number captured. In 2018, the colour pattern of the trap significantly affected the number of females captured (Table 6), with the standard trap colour combination (BBW) capturing more mosquitoes than the WWW and BWW patterns (model contrasts: *χ*^2^ > 7.97, *P* < 0.005 in both cases) (Fig. 7c). There was no significant difference between BBW and WBW traps in terms of number of females captured. There was no effect of distance to the nearest house on catch rates in either year (Table 6).

**Table 6:** Generalized linear model of the effect trap position (upwards, downwards or sideways), colour combination and distance to nearest house on the number of female *An. gambiae* (*s.l.*) captured in 2017 and 2018 in Mali

Variable	2017			2018	
	df	Likelihood ratio	P value	Likelihood ratio	P value
Trap position	1	0.288	0.592	0.146	0.702
Colour combination	3	3.831	0.280	12.771	0.005
Distance to nearest house	1	0.056	0.814	0.150	0.698

##### Proportion of females and males

There was no significant effect of the colour combination on the proportion of males captured in either year (logistic regression *χ*^2^ > 5.75, *P* > 0.125 in both cases) (Fig. 7b, d). In 2017, with no heat source, traps positioned sideways collected a significantly higher proportion of males (17.1%) than those positioned downwards (7.3%) (logistic regression: *χ*^2^ = 8.4, *P* = 0.004) (Fig. 7b). In 2018, with heat source, the number of females captured greatly increased (see previous sections) and the relative proportion of males decreased to 3.9%. The proportion of males captured was not affected by trap position (logistic regression: *χ*^2^ = 2.58, *P* = 0.108) (Fig. 7d).

##### Species composition of catches

**Fig. 8:**
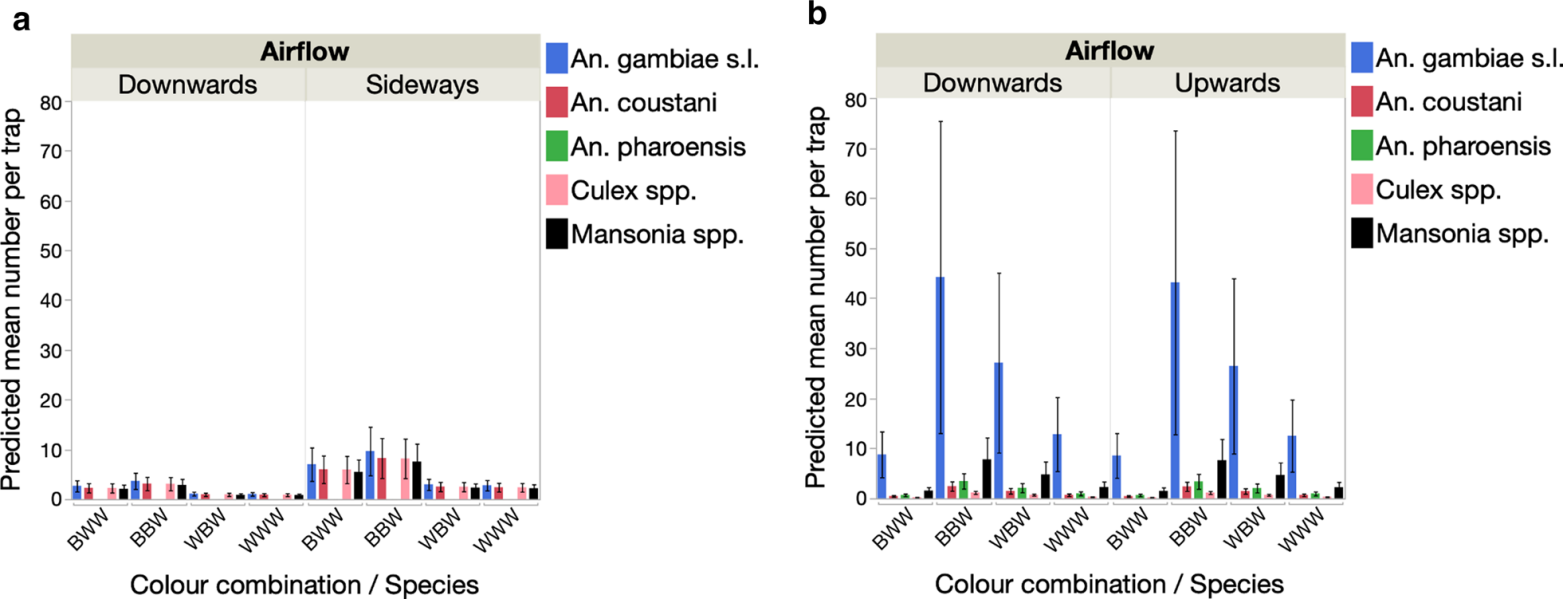
Effect of trap position and the addition of a heat stimulus on the number of females from different taxa captured in the Mali 2017 BGS (**a**) and Mali 2018 BGS with heatpad (**b**) experiments

In addition to capturing *An. gambiae* (*s.l.*) females, the BGS captured females of *An. coustani*, *An. pharoensis*, *Culex* spp. and *Mansonia* spp. The number of females captured significantly differed for each species (Fig. 8, Table 4). In 2017, the numbers were low and there were no significant differences in the number of *An. gambiae* (*s.l.*) females and females of other species captured per trap (model contrasts: *P* > 0.05 not significant [NS] in all cases). The only exception was *An. pharoensis* females, which were significantly rarer than any other species (model contrasts: *χ*^2^ > 13.25, *P* < 0.001 in all 4 comparisons). In addition, traps positioned sideways captured more mosquitoes than traps oriented downwards, and there was a significant effect of colour combination on catch rates (Fig. 8a; Table 7). Traps with the original colour combination BBW and those with the BWW combination did not differ significantly in catch rates (model contrasts: *χ*^2^ = 0.8, *P* = 0.370), but traps with the BBW combination performed better than those with the WBW and WWW patterns (model contrasts: *χ*^2^ > 6.01, *P* < 0.014 in both comparisons). In 2018, the same species were collected from traps. There was no significant difference in female catch rates between traps positioned upwards or downwards, but the number of females captured per trap strongly differed between species and colour combinations (Fig. 8b; Table 7). Female *An. gambiae* (*s.l.*) were caught in significantly greater numbers than any other species (model contrasts: *χ*^2^ > 51.0, *P* < 0.001 in all 4 comparisons). Female *Mansonia* spp. were significantly more abundant than *Culex* spp. and *An. coustani* (*χ*^2^ > 5.31, *P* < 0.022 in both cases) but not more abundant than *An. pharoensis* (*P* > 0.05 NS). Traps with the original colour combination BBW performed better than any other combination (model contrasts: *χ*^2^ > 4.78, *P* < 0.030 in all case) (Table 7), followed by WBW as second best performing combination, outperforming the BWW and WWW patterns (*χ*^2^ > 11.34, *P* < 0.001 in both cases) (Fig. 8).

**Table 7:** Generalize linear models of the effects of trap position, colour combination and species on the mean number of females from different species captured in Mali in 2017 and 2018

Source	2017			2018	
	df	Likelihood ratio χ^2^	P value	Likelihood ratio χ^2^	P value
Trap position	1	8.9	0.003	0.02	0.900
Color combination	3	11.3	0.010	38	<0.001
Species	4	19.4	0.001	260.2	<0.001

## Discussion

To the best of our knowledge, this is the first report on the impact of adding an additional heat source to the commercially available BGS trap, a trap used for *Aedes* surveillance in many parts of the world. Across two studies conducted in the Sudano-Sahelian zone of western Burkina Faso and west-central Mali, the BGS traps with the addition of a heatpad outperformed the standard trap, resulting in approximately a two- and sixfold increase, respectively, in anopheline catch rates. By comparison, changing the position of the trap from upwards to downwards or sideways and/or manipulating its pattern of colour contrast had much less impact on the capture efficacy. In terms of capture rate, the standard upward positioning and the standard colour configuration were the best configuration when combined with the heat stimulus, as this configurationt led to high specificity for female anopheline malaria vector species across both locations.

The 2016 Burkina Faso experiment assessed the impact of trap position and the addition of a heat source to the BGS traps on catch performance. The results demonstrated that the addition of a heat source to BGS traps clearly improved their performance. Interestingly, changes in trap positions led to differences in catch rates only when the heat source was present, with BGS in the upwards position then performing the best. These results are in contrast to those reported from studies that focussed on *An. darlingi* in South America and *An. arabiensis* in Tanzania, which reported higher catch rates with a downward trap orientation [17, 19, 20].

In addition to *An. gambiae* (*s.l.*), which dominated the catches, a number of other mosquitoes were caught, including other anophelines, *Culex* spp. and *Mansonia* spp. Aedine mosquitoes are sometimes found at the study site, but none were caught in our study, probably due to the nocturnal sampling strategy used. In its best configuration, the BGS in an upwards orientation with the addition of the heat stimulus (BGSH) captured approximately twice as many individuals of all taxa compared to the configuration without heat pad. *Mansonia* spp., an exophillic and zoophilic species, was second in abundance. The BGSH captured approximately 100 female *An. gambiae* (*s.l.*), the main malaria vector in the area [25, 27] per night despite being set in an area relatively distant from habitations (about 200 m). The BGSH also captured good numbers of the secondary malaria vector *An. pharaoensis* and *An. coustani*, two exophillic and zoophillic species, which are rarely caught with other surveillance methods [26, 28]. The high specificity of the BGSH for malaria vectors observed in our study and the high numbers caught are in contrast with previous studies focusing on BGS trap configurations conducted without additional heat. In a study carried out in Tanzania [20], the anopheline catch ratio was approximately 10%, with the main part of the catch being *Culex* spp. [20]. In a previous study carried out in Burkina Faso, a high proportion of anophelines was obtained in BGS traps set in outdoor settings, but the overall catch rates were very low [18].

The aim of the Mali 2017–2018 experiments was to assess the impact of different trap colour contrasts in addition to testing trap orientation and the addition of a heat source. In both years, BGS traps collected a similar range of species as that found in the Burkina Faso study (*An. coustani*,* An. pharoensis*,* Culex* spp. and* Mansonia* spp.), and catches were dominated by female *An. gambiae* (*s.l.*). Baseline indoor female densities, as measured by PSC, were similar in both years, but the addition of the heat source in 2018 dramatically increased the capture rate by over sixfold. That the BGSH catch rates did not depend on the distance to the nearest occupied house suggests that the traps are attractive to *An. gambiae* (*s.l.*) in the absence of human hosts. Furthermore, the mean catch rates in BGSH traps were comparable to that of PSC, which is one of the most effective methods of indoor mosquito collection [10, 11]. As in Burkina Faso, varying the trap orientation downwards versus sideways and upwards versus downwards only impacted catch rates when a heat source was added. As discussed previously, the better performance of the BGSH positioned upwards in in contrast with previous studies that focused on comparisons of the BGS upwards versus downwards [17, 19, 20]. At present, it is not possible to determine whether these differences result from the different geographical and ecological settings in which they were conducted, or from differences in the mosquito species present.

Mosquitoes use olfaction and vision to locate their hosts over long distances [36]. In the Mali studies, in addition to the trap orientation and heat, the colour patterns of trap significantly affected the number of females captured. Of the different colour combinations tested, the BBW standard trap performed the best. Thus, there remains some ambiguity about the visual preferences of *Anopheles*, with some studies indicating a preference for black traps [37] and others for white ones [17]; this study highlighted the efficacy of the standard BBW BGS pattern (dark body with white top).

Even though the results obtained from the studies carried out in Burkina Faso and Mali are consistent in terms of the importance of trap orientation and heat source, a large disparity was observed in the magnitude of the enhancing effect of the heat pad. In fact, while the Burkina study showed a twofold enhancing effect, this was as much as sixfold in Mali. However, the overall catch rates were lower in Mali, with approximately 60 mosquitoes caught per trap per night compared to over 200 in Burkina Faso [25, 26]. Therefore, one possible explanation for the discrepancy in the enhancing effect of the heatpad would be that the BGS trap performance decreases beyond a particular threshold number of mosquitoes caught because they slow the aspiration through the catch bags and decrease its performance. Another important difference between both experiments was how the heat pads were used. In the Burkina study, heat pads were stuck on the inside wall of the BGS traps. In the Mali study, heat pads were stuck on the exterior surface of the white top of BGS traps. This difference may have led to different patterns of heat dispersion. In the Malian BGSH traps, the position of the heat pad resulted in the heat source being visible on the outside of the trap, perhaps promoting its attractiveness.

In addition to further studies aimed at understanding the effect of heat modulation on mosquito catch dynamics, future work should focus on improving other characteristics of the BGSH. To fit the requirements of large-scale surveillance programmes, it would be particularly important to provide an enhanced CO_2_ supply and heat source that would allow the BGSH trap to remain effective over several days of continuous trapping rather than the current 12 h. Additional studies are also necessary in order to assess the BGSH trapping effectiveness in unfavourable conditions such as low mosquito density environments or areas further away from human settlements.

## Conclusion

The increasing number of reports that indicate mosquito populations are resistant to most insecticides used for public health and show high levels of exophilly and exophagy calls for novel monitoring and control tools [9]. Results from the studies carried out in the two West African villages reported here indicate that adding a heat source to a standard BGS trap in an upwards configuration (and supplied with a CO_2_ source) can strongly improve its catch rate. This trap configuration, referred to as BGSH, is particularly effective for capturing females of *An. gambiae* (*s.l.*) and important secondary vector species, such as *An. funestus*, *An. coustani* and *An. pharaoensis*. Catch rates were improved several-fold when heat was added, and the traps remained effective even at a distance of several hundred metres from houses. The BGSH offers the possibility of scalable standardized sampling of increasingly exophagic and exophillic malaria vector populations and may provide novel opportunities for disease vector surveillance and monitoring geared specifically towards sampling outdoor anopheles populations.

## Data Availability

The datasets generated and/or analysed during the current study are not publicly available due to the fact that they are part of a larger research project that is still ongoing; but are available from the corresponding author on reasonable request. Nevertheless, all data important for the understanding of the described results and conclusion are directly included in tables or figures within the manuscript.

## Abbreviations

CDC: Centers for Disease Control and Prevention
EDSM-V: Cinquième Enquête Démographique et de Santé du Mali
HLC: Human landing catches
IRSS: Institut de Recheche en Sciences de la Santé
PSC: Pesticides spray catch
